# The national child odontology registry (SCOR): a valuable resource for odontological and public health research

**DOI:** 10.1186/s12903-023-03199-1

**Published:** 2023-08-29

**Authors:** Nikoline Nygaard, Lars Ängquist, Daniel Belstrøm, Evelina Stankevic, Torben Hansen, Anja Olsen, Kasper Rosing, Merete Markvart

**Affiliations:** 1 Department of Odontology, Section for Clinical Oral Microbiology, Faculty of Health and Medical Sciences, University of Copenhagen, Nørre Allé 20, Copenhagen, 2200 Denmark; 2 Novo Nordisk Foundation Center for Basic Metabolic Research, University of Copenhagen, Blegdamsvej 3b, Copenhagen, 2200 Denmark; 3 Danish Cancer Society Research Center, Nutrition and Biomarkers, Strandboulevarden 49, Copenhagen, 2100 Denmark; 4 Department of Odontology, Section for Community Dentistry, Faculty of Health and Medical Sciences, University of Copenhagen, Nørre Allé 20, Copenhagen, 2200 Denmark

**Keywords:** Dental caries, Gingivitis, Periodontitis, Oral health, Registries

## Abstract

**Background:**

Since 1972 The National Child Odontology Registry has collected data on the oral health of most of all Danish children and adolescents. However, comprehensive information on the registry has not previously been available, making it difficult to approach and use the registry for research purposes.

**Methods:**

By combining historical documentation and simple descriptive statistics we provide an overview of major events in the timeline of The National Child Odontology Registry and discuss how they impact the available data. We provide a broad overview of the dental variables in the registry, and how the registration criteria for some of the core dental variables (gingivitis, periodontitis, and dental caries) have changed over time. We then provide examples of how aggregate variables for the core dental diseases, allowing for comparison across registration criteria, can be created.

**Results:**

Most of the Danish population born during or after 1965 have a least one entry in the National Child Odontology Registry, with 68% having entries spanning their entire childhood and adolescence. The prevalence of gingivitis and periodontitis seem to increase significantly in the years immediately following changes in how registration criteria for these variables, raising questions as to whether these diseases are generally underreported, or subject to overreporting in the years following the registration changes. The mandatory ages of registration instituted in 2003, do not appear to have had a strong impact on the ages at which registrations are made. For variables not directly comparable across datasets due to changes in registration criteria aggregate variables of measurements can be computed in most cases.

**Conclusions:**

The National Child Odontology Registry provides a unique opportunity to study the impact of childhood oral health on life trajectories, but using the registry is not without issues, and we strongly recommend consulting with experts in the field of odontology to ensure the best use of available data.

**Supplementary Information:**

The online version contains supplementary material available at 10.1186/s12903-023-03199-1.

## Introduction

The National Child Odontology Registry (SCOR) was established in 1972 to record the oral health of Danish children and adolescents on an annual basis [1]. The registry was constructed for both epidemiologic and administrative purposes and is based on registrations from the Danish Child Oral Healthcare Services (DCOHS), which offers tax-financed dental care for all Danish children and adolescents [1].

Since 1972, successive generations of Danish children have been enrolled into the DCOHS, and thus entered SCOR. Data from SCOR can be combined with data on every contact an individual has with the public healthcare and welfare systems in Denmark. This can be done using the personal identification numbers assigned to each Danish citizen, enabling the use of SCOR as a nationwide, longitudinal cohort with continuous enrollment and follow-up. As such, SCOR potentially makes for a comprehensive and continuously expanding registry of oral health for most of Danish children from 1972 to today, and therefore a unique resource for both cross-sectional and longitudinal studies of oral health. However, SCOR has hitherto not been extensively utilized for research purposes and is neither well known nor well understood outside of the field of odontology in Denmark.

We, therefore, aim to investigate whether SCOR can be useful for research purposes, by examining the contents of SCOR, investigating the consistency of data over time through descriptive statistical analyses, and testing the feasibility of constructing core dental variables spanning the entirety of SCOR. Furthermore, we aim to provide a summary of available documentation on the changing registration practices and guidelines throughout the years of SCOR and to give recommendations for best practices, when using the database for research beyond the field of dentistry.

## Materials & methods

### Historical documentation

To create an overview of the changes made to SCOR in terms of registration requirements and practices we compiled information from laws with relevance to SCOR [2–4] and guidelines made by the Danish Health Authority [5–9]. Most of this material we received directly from the Danish Health Authorities, but it can also be assessed via the National Archive [10]. Material not received from the Danish Health Authorities was found on freely accessible websites with all laws and proposed laws passed in Denmark [11, 12]. An overview of major events in the timeline of SCOR can be found in Supplementary figure file 1.

### SCOR data

SCOR contains data from routine examinations of the dental health of a majority of Danish children and adolescents. A centrally created purpose-made registration form has been used for this during the entirety of SCOR’s existence, though it has changed over time, as the requirements for what is to be reported have changed. The registration form began as a preprinted paper form, filled out by hand by a dentist, dental hygienist, or dental assistant based on examinations conducted by dentists, and scanned at a central archive [1]. Today registration forms are filled in and filed digitally.

Between 1972 and 1976, 109 out of 277 municipalities held a dispensation from the requirement of reporting to SCOR [4, 13]. Thus, SCOR did not gain nationwide coverage until 1977. Additionally, a few of the municipalities listed in SCOR, accounting for only a very small number of registrations, have never existed. These nonexistent municipalities are likely a result of the original optical character recognition registration forms requiring municipal codes to be filled in by hand, leading to errors upon scanning.

During the first decades of SCOR´s existence, yearly reports were mandatory, including at least one report on every individual under the scheme, i.e. all individuals in all age groups were to be reported each year. Initially, the “year of report” followed the school year (beginning and ending at the summer holidays). From 1987, years of report were reset to start by the date of birth. To facilitate this switch, it was not mandatory to report visits to the DCOHS from the 1st of August 1987 to the 1st of January 1988. In 1993, in response to the vastly improved dental health status of Danish children, it was decided that only data from four points in time, by age 5, 7, 12, and 15 years, during an individual’s period of eligibility for care in the DCOHS, should be subject to mandatory reporting to SCOR. However, municipal dental services are free to choose to register theoral health status of all age groups with the purpose of more extensive monitoring of child oral health status within the municipality [7]. In 2013, a system for needs-based graduation in the frequency of visits was implemented, officially allowing for up to 24 months between visits, so long as the mandatory reports at ages 5, 7, 12 and 15 years are made [14]. However, as we will see, the implications of something being mandatory in SCOR are not entirely clear.

SCOR contains approximately 350 variables, covering dental caries, pathological pockets (a proxy for periodontitis, henceforth periodontitis will be used in this paper), gingivitis, trauma, tooth position, and jaw deviations. A full list of the variables included in SCOR can be found on the website of the Danish Health Data Authority [15]. For the present study, all variables in SCOR relating to dental caries, gingivitis, and periodontitis for all individuals enrolled between 1972 and 2010 were included, with data from follow-up ending in 2021.

### Other registry data

For this paper, data from SCOR was combined with data on date of birth and where applicable year of death for all individuals in SCOR from Statistics Denmark (DST).

To approximate the number of individuals eligible for care in the DCOHS, we used freely available data on the number of live births in Denmark during the period 1965–2010 [16], and the number of people between the ages of 0–18 who were granted Danish citizenship to Denmark in the period 1979–2010 [17] from DST.

### Creation of core dental variables from SCOR data

SCOR comes in three different datasets, 1972–1987, 1988–1994, and 1995 – current day. The division of these datasetsdoes not correspond entirely with changes in registration criteria (Tables 1 and 2 and Supplementary File 2). In addition, not all variables appear in all datasets, while other variables appear in several datasets, but with different implications. Crucially core dental variables for gingivitis, periodontitis, and dental cavities are not directly comparable across datasets, necessitating the creation of aggregate (derived) variables spanning all three datasets.

**Table 1:** Registration of gingivitis in SCOR, 1972–2022 using Fédération Dentaire Internationale (FDI) notation

1972–1987		1988–1999		2000-present	
1	Slight changes in the color and surface of gingiva, no bleeding on probing.	1*	Mild gingivitis – initial changes in the margo gingiva and at the top of the gingival papillae, no bleeding on probing.	X	Missing registration tooth (lost or not yet erupted).
2	Moderately inflamed gingiva, changes in color, shape and surface, bleeding upon probing.	2	Moderate gingivitis – bleeding upon probing at 0.5 mm beneath margo gingiva.		
3	Severe inflammation, clear changes in the color, shape and surface of the gingiva, ulceration.	X	Missing registration.		
Mandatory registration.		Mandatory registration from age 7.		Non-mandatory registration.	
Registered on 4 index teeth (tooth 52 and 55, and 82 and 85 in the temporary dentition, and 12 and 16, and 42 and 46 in the permanent dentition). If a registration tooth is missing the nearest neighboring tooth is used. Calculated as index.		Registered on 12 index teeth (teeth 11, 12, 21, 22, 31, 32, 41, 42 and 16, 26, 36, 46). Calculated as index.		Registered on 12 index teeth (teeth 11, 12, 21, 22, 31, 32, 41, 42 and 16, 26, 36, 46). Calculated as index.	

*Non-mandatory registration

**Table 2:** Registration of periodontitis in SCOR, 1972–2022 using Fédération Dentaire Internationale (FDI) notation

1972–1987		1988–1999		2000-present	
NA	Either no pathological pockets or no registration.	Blank	Pockets < 4mm.	1	Clinical attachment loss at minimum one site upon probing along the cervical line (Periodontitis marginalis).
X	Is registered if the junctional epithelium for one or more teeth is located apically to the cemento-enamel junction.	4	Pockets > 4mm.	X	Missing registration tooth (lost or not yet erupted).
		5	Pockets > 4mm with attachment loss and the bottom of the pocket lying apically to the cemento-enamel junction.		
		X	Missing registration tooth.		
Non-mandatory registration.		Mandatory registration from age 14.		Mandatory registration for ages 12 and 15.	
Registered on 4 index teeth (tooth 52 and 55, and 82 and 85 in the temporary dentition, and 12 and 16, and 42 and 46 in the permanent dentition). If a registration tooth is missing the nearest neighboring tooth is used.		Registered on 12 index teeth (teeth 11, 12, 21, 22, 31, 32, 41, 42 and 16, 26, 36, 46). Calculated as index.		Registered on 12 index teeth (teeth 11, 12, 21, 22, 31, 32, 41, 42 and 16, 26, 36, 46). Calculated as index.	

In Table 3, we provide an example of how to use the data available in SCOR to create aggregate variables allowing for comparison across registration approaches for gingivitis, periodontitis, and caries measured by the number of “decayed, missing, filled” surfaces (DMF). The severity of dental caries was calculated as the sum of the decayed surfaces with codes 1, 2, 4, and 6 at each visit (Supplementary Table 1). For the aggregate variables for gingivitis and periodontitis, we decided that these should indicate a general oral condition, rather than for example in the case of gingivitis, random bleeding by a few teeth, as frequently occurs even in healthy individuals. For this purpose, cut-off points were set based on a dental professional assessment. For the period 1972–1987, individuals were classified as having gingivitis if they had a gingivitis score of 9 or more, meaning that at least one out of four registration teeth were registered with severe gingivitis (Table 1). To be classified as having periodontitis only required one tooth registered with pathological pockets, as this was a non-mandatory registration at the time, and w, therefore, reasoned that the registration being made would be indicative of a significant level of disease. From 1988 and onwards, we grouped individuals as having gingivitis or periodontitis if at least 6 out of 12 registration teeth were registered as showing gingival bleeding upon probing or pathological pockets, respectively, which we judged to indicate a state of general disease.

**Table 3:** Calculation of aggregate variables for gingivitis, periodontitis and caries (DMF) spanning all registrations

		1972–1987	1988–1999	2000–2021
Gingivitis	Yes	Gingivitis index ≥ 9	Gingivitis level ≥ 6	Gingivitis level ≥ 6
	No	Gingivitis index < 9	Gingivitis index < 6	Gingivitis index < 6
Periodontitis	Yes	Pathological pockets = X	Registered pockets ≥ 6	Registered pockets ≥ 6
	No	Pathological pockets = NA	Registered pockets < 6	Registered pockets < 6
Caries (DMF)	Temporary dentition (dmf_s)	Sum of surfaces with caries codes 1, 2, 4 or 6 (flade_1_1, flade_2_1, flade_4_1, flade_6_1)	Sum of surfaces with caries codes 1, 2, 4, 5 or 6	Sum of surfaces with caries codes 1, 2, 4, 5 or 6
	Permanent dentition (DMF_S)	Sum of surfaces with caries codes 1, 2, 4 or 6	Sum of surfaces with caries codes 1, 2, 4 or 6	Sum of surfaces with caries codes 1, 2, 4 or 6

#### Notes on the gingivitis aggregate variable

Gingivitis index, *gingivitisindex in SCOR*, was calculated by grading 4 index teeth on a scale from 0 to 3, resulting in a score of 0 to 12 (Table 2: Column 1 for the meaning of grades 0 to 3). Gingivitis level is the number of registration teeth (N = 12) with code 2 (Table 1: Column 2), resulting in a score of 0 to 12 between 1988 and 1999, *gingiv2* in SCOR. Since the year 2000, gingivitis level is based on the variable *gingiv1* in SCOR,, as since the turn of the century this have been the only variable for registering gingivitis, encompassing all cases of gingivitis regardless of severity (Table 1: Column 3). It is still applied to 12 index teeth and results in a score of 0 to 12. The denotation used for registration teeth in SCOR is the Haderup system. For the convenience of an international audience, we have converted this to the Fédération Dentaire Internationale (FDI) system in this article. **Notes on the periodontitis aggregate variable**: The variable pathological pockets, *patologiske_pocher in SCOR*, denotes whether any of the registration teeth were given registration X (Table 2: Column 1), while registered pockets, *forstet5 in SCOR*, denotes the number of registration teeth with code 5 (Table 2: Column 2) between 1988 and 1999. From the year 2000 onward, it is necessary to calculate the number of teeth with a periodontitis registration by summing registrations of pathological pockets (Table 2: Column 3) for the 12 registration teeth (*pockets = poch11_51 + poch12_52 + poch16_56 + poch21_61 + poch22_62 + poch26_66 + poch31_71 + poch32_72 + poch36_76 + poch41_81 + poch42_82 + poch46_86*). **Notes on the caries aggregate variables**: From 1988 onwards, variables for DMF are provided in SCOR. According to the available documentation from The Danish Health Data Authority, these variables are calculated from the number of surfaces with codes 1, 2, 4, 5, and 6 (see Supplementary Table 1 for the meaning of codes). The internationally used DMF variables do not include code 5, and thus we opted to omit this code and to recalculate variables for the entirety of SCOR to facilitate comparison with international data.

### Statistical analysis

Prior to statistical analysis, data files were merged and cleaned. All duplicate entries were removed, as were the faulty entries e.g. date of death prior to the date of examination, and registrations of ages outside of what qualifies for care under the DCOHS (0 < age < 19 years). For ease of analysis and interpretation we restricted the dataset to one entry per year of age (0, 1, 2, …, 18) per individual. For this purpose, age at visit was calculated as the difference between the date of visit and date of birth and selecting one entry per individual at a given age, using the earliest entry at a given age rounded to integer, for inclusion in the dataset. The minimum and maximum number of entries for each individual, along with average number of entries and the standard deviation, were calculated. To estimate the possibility of following individuals over time in SCOR we calculated the proportion of individuals with reports during one to four different times during their eligibility for care in the DCOHS (age 0–6, 7–11, 12–14, and 15–18 years).

To evaluate the validity of data, we compared the number of individuals in SCOR by birth year, with the number of births and immigrants granted citizenship in the corresponding years as registered by DST. The difference between DST and SCOR data were calculated in absolute numbers and percentages, by year and as a total of the entire period.

To examine the consistency of data over the span of SCOR the number of individuals with at least one entry into SCOR by year of visit and year of report was plotted. As the variable ‘year of report’ disappears from SCOR with the registration changes in 1999–2000, we used the year of visit as a proxy for the year of report for the years 2000 and onwards. Additionally, the percentage of individuals with a least one report at a given age before and after the mandatory ages of report were implemented in 1993, were calculated.

To examine the reliability of the aggregate gingivitis, periodontitis and caries variables, we plotted the prevalence by sex and at ages 5, 9, and 15 (chosen to reflect the temporary, mixed and permanent dentition) across the span of SCOR. To plot caries the variables for temporary (*dmf_s*) and permanent (*DMF_S*) dentition at a given visit were summed, and this value used.

To gauge whether individuals with oral disease measured by the aggregate gingivitis, periodontitis and caries are over- or underrepresented in SCOR compared to the disease free population, we plotted the mean number of entries by disease state. In the case of caries, the two variables denoting DMF in the temporary and permanent dentition respectively were summed, and the highest number of affected surfaces at any point in time for each individual chosen. Based on this value individuals were grouped in intervals of 0–4, 5–9, and ≥ 10 affected surfaces, in accordance with grouping used by the Danish Health Authorities in their analysis of a corresponding SCOR (*DMF_S*) variable [18].

All analyses were performed using R (v.4.2.1.) with Rstudio (IDE version 2022.07.2 + 576).

### Ethics approval

The use of registry data in this study was approved by the Danish Data Protection Agency via the Office of Research and Innovation, University of Copenhagen, case number 514 − 0496/20–300.

## Results

### Inclusion into SCOR

From 1972 and the following 10 years, the DCOHS utilized a gradual enrollment strategy, beginning with all children aged 7 in 1972. In 1973, the new generation of 7-year-olds was included, in 1974 the next generation of 7-year-olds, and so forth until, eventually, all age groups from 7 to 16 were covered (with the 16-year-olds in 1981 being those who were included at the age of 7 in 1972) [2]. From 1981, children under the age of 7 were gradually included into the DCOHS [4], and in 1986, the DCOHS further expanded to include all children and adolescents up till the age of 18 [3]. However, SCOR data shows that in practice a broad range of ages has been included since 1972 (Supplementary Fig. 3).

### Validity of the available data sample

**Fig. 1:**
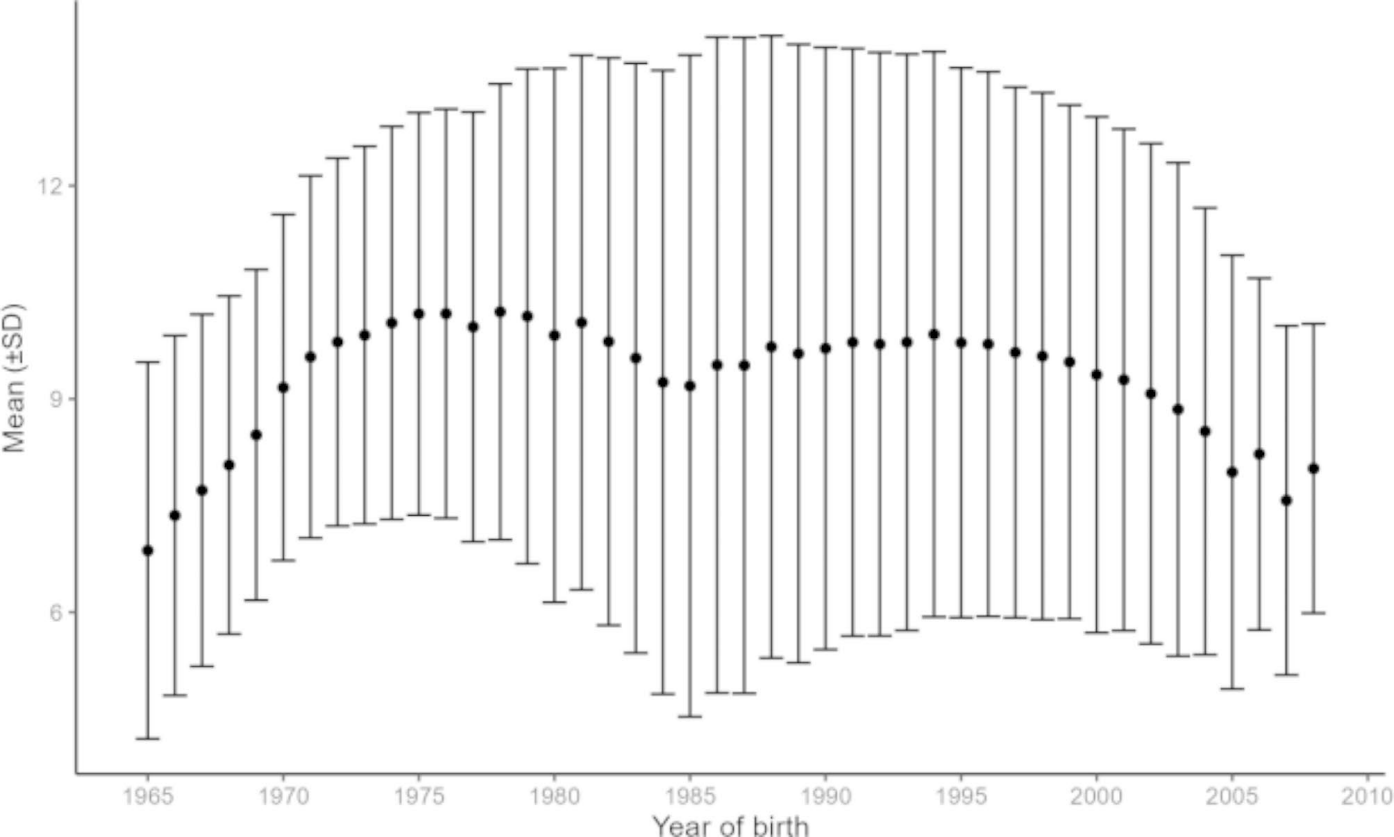
Mean entries in SCOR per individual by birth year. Mean and standard deviation (SD) of the number of entries in SCOR per individual by birth year

A total of 4,012 duplicate entries were removed, along with 14,860 entries considered faulty entries. Of the faulty entries removed, 67% (9,995 entries) stemmed from the first 15 years of SCOR data. An additional 2,155,897 (7.9%) of entries were removed in restricting the dataset to one entry per individual per year of age. The resulting dataset consisted of 2,968,331 individuals with a total of 24,988,781 entries. The minimum number of registered visits per individual is one, and the highest 31, with an average of 9.14 and 8.42 entries per individual, with no apparent differences between males and females for either dataset (Table 4). Mean number of entries by birth year and their standard deviations are provided in Fig. 1. Looking at the spread of visits across the time eligible for treatment in the DCOHS grouped by age (0–6, 7–11, 12–14, and 15–18 years), 83.1% of the individuals in SCOR have entries recordedduring three or four different periods in time during eligibility for care in the DCOHS, with no significant difference between males and females (Table 4).

**Table 4:** SCOR population characteristics

	Females	Males	Total
N (%) of individuals in SCOR	1,448,756 (48.8)	1,519,575 (51.2)	2,968,331 (100)
N (%) of entries in SCOR	12,223,687 (48.9)	12,765,094 (51.1)	24,988,781 (100)
Mean (SD) number of entries per individuals	8.44 (3.32)	8.40 (3.32)	8.42 (3.32)
Minimum; maximum entries per individual	1; 18	1; 18	1; 18
N (%) of individuals with entries in SCOR recorded at			
Four different periods in time	748,669 (51.7)	776,772 (51.1)	1,525,391 (51.4)
Three different periods in time	456,652 (31.5)	482,882 (31.8)	939,534(31.7)
Two different periods in time	176,678 (12.2)	189,248 (12.5)	365,926 (12.3)
One period in time	66,746 (4.6)	70,714 (4.7)	137,460 (4.6)

SD = standard deviation

There are more people registered in SCOR compared to DST when looking at the number of children born between 1965 and 2008. Please note that because our dataset only includes individuals having been registered for the first time in SCOR up untill 2010, we have no data on those born in 2009 and 2010, as they had not been enrolled into SCOR in 2010 due to their young age. However, when considering immigration, measured by individuals granted permanent residency, there are 3.35% fewer children registered in SCOR compared to DST (Supplementary Table 3). The difference between the number of individuals registered in SCOR and DST is likely due to individuals born after 2005 in many cases being too young to have a reported visit with the DCOHS, as they would have been aged 4 at the most in 2010 when inclusion into the data for this study ends. When restricting the comparison to individuals born between 1965 and 2005, the difference drops to -0.24 (Supplementary Table 3). See Supplementary Table 3 for data on the differences between SCOR and DST data by individual birth years.

**Fig. 2:**
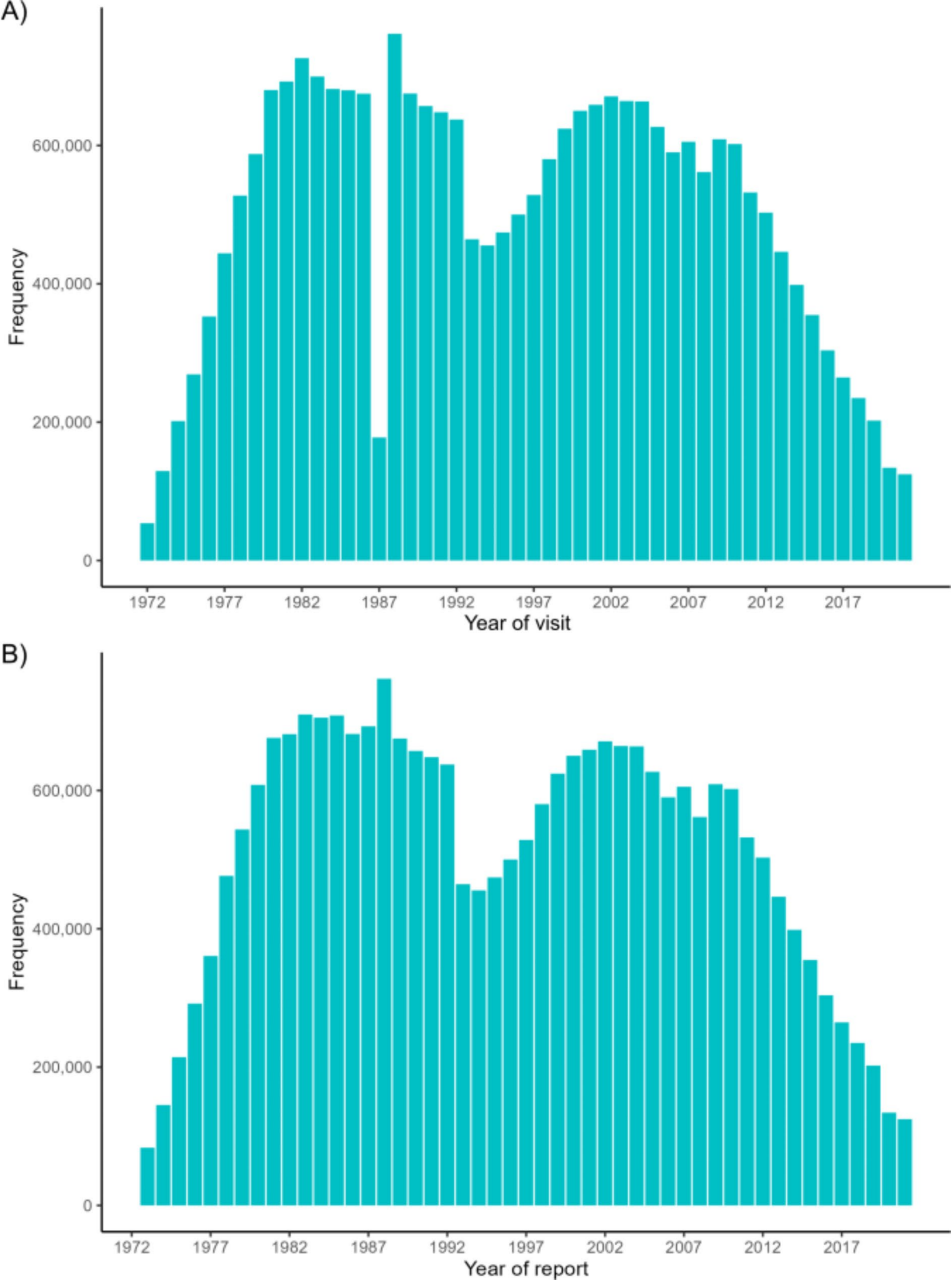
Total number of registered visits by year of visit and year of report. Total number of registered visits by year of visit (A) and year of report (B). See Supplementary Table 2 (A) and Supplementary Table 4 (B) for the numbers underlying the figure

In the years 1987–1988, registrations switched from following the school year (beginning and ending at the summer holidays in June-August), to following individual birth years. This meant that from the 1st of August 1987 to the 1st of January 1988 registrations were not mandatory, which is reflected in the severe drop in registrations seen in 1987 (Fig. 2: Panel A). A similar drop in registrations is not seen for the year of report (Fig. 2 panel B), which have always run from January 1st to December 31st. Note that the two variables contain the same amount of data, albeit distributed differently across the years of SCOR.

**Fig. 3:**
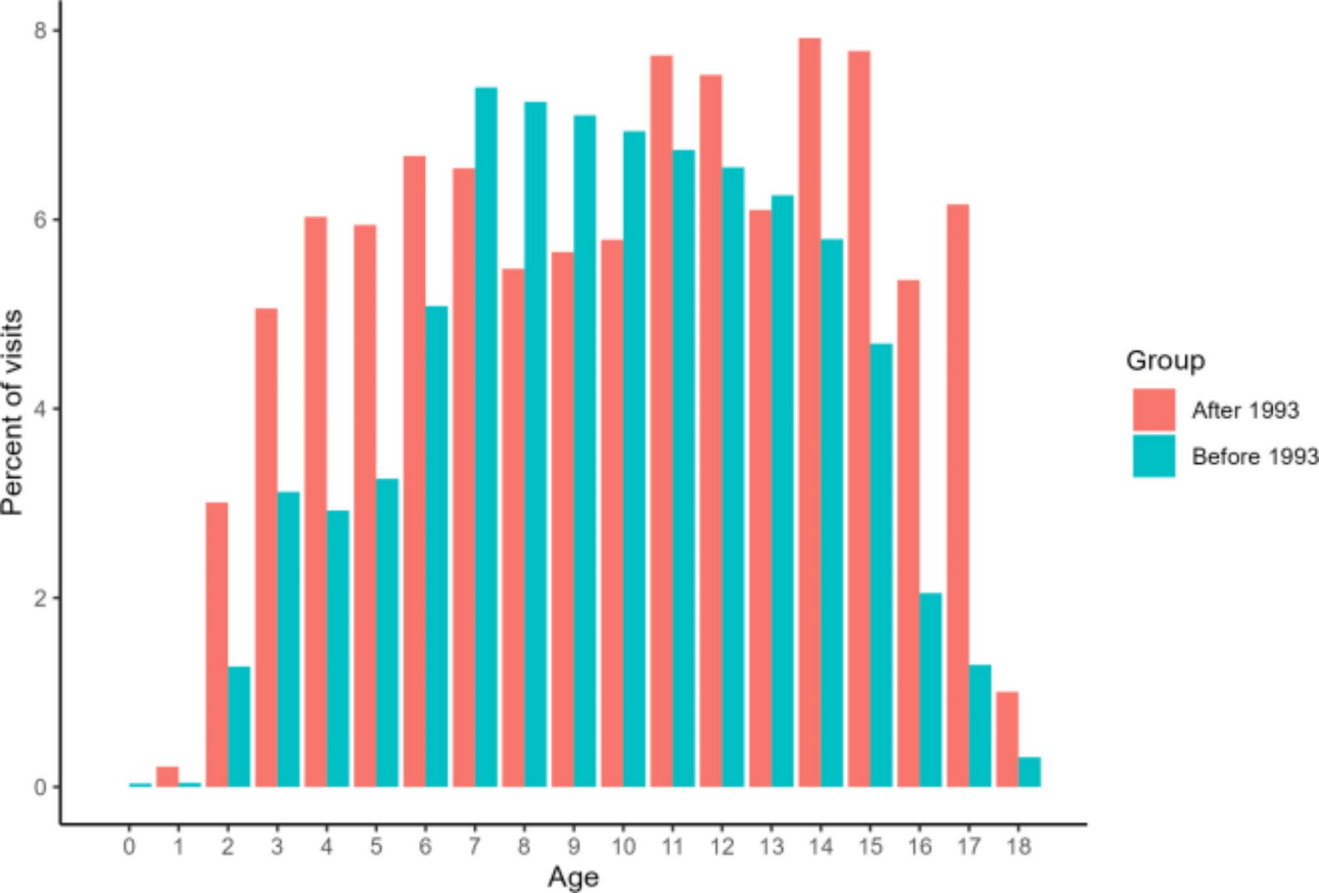
Number of visits by age before and after 1993. See Supplementary Table 2 for the numbers underlying the figure

A decrease in the number of visits for all ages by both year of visit and year of report is seen in 1993 (Fig. 2). Reports to SCOR appear to be somewhat more evenly distributed across different ages after the implementation of the mandatory ages of report in 1993 (Fig. 3). After 1993 reports are not limited to the mandatory ages of report as one might expect, in fact more individuals are reported to have a visit at ages 6, 11, and 14 than ages 5, 7, 12, and 15 (Fig. 3).

### Construction and contents of SCOR

**Fig. 4:**
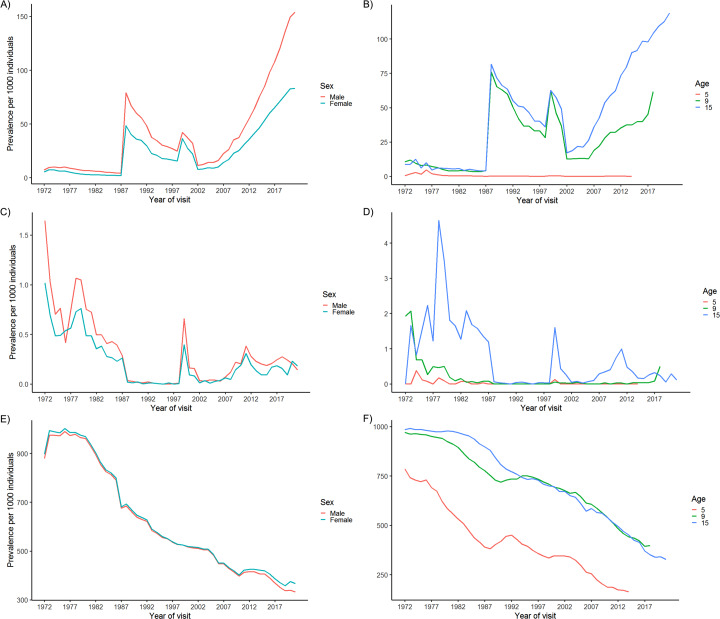
Prevalence of gingivitis periodontitis, and caries by sex and age. Prevalence of gingivitis (A-B), periodontitis (C-D) and caries (E-F) by sex and age. Prevalence per 1000 individuals by sex (A, C and E) and at ages 5, 9, and 15 (B, D and F) for gingivitis (A and B), periodontitis (C and D), and caries (E and F)

**Fig. 5:**
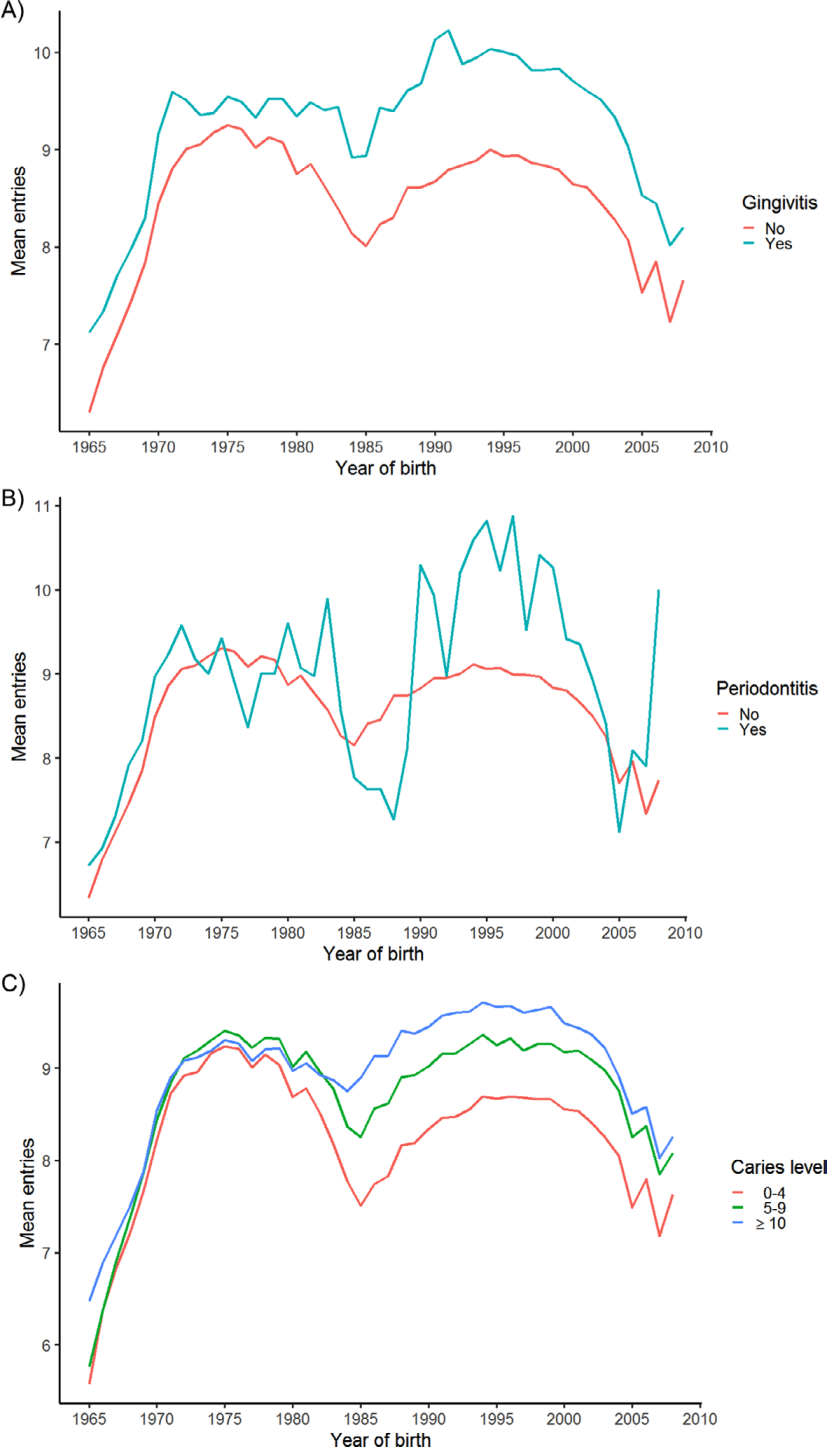
Mean entries in SCOR by presence/absence of gingivitis, periodontitis, and caries level. Mean entries in SCOR by presence/absence of gingivitis (A), periodontitis (B) and caries level (C)

A suggested method for creating aggregate measures for the core dental variables gingivitis, periodontitis, and caries were presented in Materials & Methods. Using these aggregate variables, it is possible to plot, for example, the corresponding prevalence over time. The prevalence graphs for both the gingivitis and periodontitis variables display marked increases in the years 1987–1988 and 1999–2000 (Fig. 4: Panel A-D), while the graphs for caries do not display similar spikes (Fig. 4: Panel E-F). Overall males have a marginally higher prevalence of gingivitis compared to females, while females appear to have a marginally higher prevalence of caries compared to males. The prevalence of gingivitis, periodontitis, and caries all increase with increasing age, with the largest increase in prevalence for gingivitis and periodontitis occurring between ages 5 and 9, and for periodontitis between ages 9 and 15. Looking at the mean number of visits by disease state, it appears that orally healthy individuals generally have fewer reported visits the DCOHS, than do diseased individuals. The difference between groups is generally small (Fig. 5), albeit with some variation in which groups has the most entries on average over the years (Fig. 5).

## Discussion

A strength of SCOR is its large sample size, breadth of registrations, and long follow-up. Despite plans for gradual enrollment, differing rules on who is to be registered, and which age groups have been eligible for care a given year (Supplementary Fig. 2), entries for all age groups except those below the age of one appear across all years (Supplementary Fig. 3 and Supplementary Table 2). We have no clear explanation as to why individuals below 1 year of age have been registered in SCOR for a brief period only, between 1975 and 1987. It is possible that dentists simply stopped reporting visits to the DCOHS by children below one year of age, as registering dental health on children still teething is for the most part of little clinical use. More than half, 83.1% of the study population, have entries spread well out across their time in SCOR, allowing for analysis of dental health across childhood and adolescence (Table 4). There is a tendency toward a slight decrease in the average number of entries per individual in the latter years of SCOR (Fig. 1). This may in part be due to individuals enrolled since 2005 not yet (in 2021) having reached the age of 18, meaning that a full set of data was not available for these individuals. Additionally, the relaxed mandatory ages of report, though they do not appear to be closely followed (Supplementary Fig. 3), may also contribute to the decreasing number of visits. Another possibility is that improved oral health in the general population allows for longer intervals between visits to the dental clinic, up to 24 months [19], resulting in fewer entries in SCOR.

The difference between the observed number of children registered in SCOR, and the expected number of children based on number of births and children granted Danish citizenship as registered by DST (Supplementary Table 3), is minor. Indeed, when restricting data to births in the period 1965–2005 there are 0.23% more individuals registered in SCOR than by DST. Other than erroneous registration of personal identification numbers, this may be attributed to children with temporary residence also being included in SCOR at times. On the other hand, individuals being granted citizenship late in adolescence may not enter SCOR. Duplicate entries are expected not to occur due to the Danish Central Person Register, which meticulously tracks the distribution of personal identification numbers, which all individuals living in Denmark are required to have, and which are used in all interactions with state-run institutions such as the healthcare system. Regardless, given the small differences between DST and SCOR data, we consider the population included into SCOR as being largely representative of the Danish population at least until 2005. This is further supported by the fact that the DCOHS operates on a free-at-the-point-of-delivery basis. It seems feasible to assume that most invitees will accept the offer of free child dental care. Yet some groups may still be underrepresented in the data. For example, people lacking Danish language skills may not respond to invites from the dentist. Unfortunately, we are not in possession of the data needed to determine the number of children in Denmark who never access care in the DCOHS. However, dentists in the DCOHS are obliged to ensure that every child connected to their clinic is seen. If a child does not attend routine examinations and follow instructions for oral care, social services are ultimately contacted [20], which is why we expect most children to access care in the DCOHS at some point. A possible barrier to entry could be the geographical location of dental clinics. Dental clinics under the DCOHS are often located in or just by schools, making it easy to attend regular examinations even without the attendance of a guardian (provided the guardian has given permission for this beforehand). It is possible though that individuals in remote areas without dental clinics situated at the local school, will be somewhat underrepresented in the data.

Some limitations appear when examining SCOR. Crucially, data seems to be greatly influenced by changes in registration practices. This is seen in the severe drop in reported visits for the years 1987–1988, when year of visit changed from following the school year to the year of birth (Fig. 2: Panel A). A similar drop is not seen for the variable year of report (Fig. 2: Panel B), which correspondingly has not undergone any changes. In both panels the implementation of the mandatory ages of report in 1993 seems to cause a decrease in overall registrations, albeit not to the extent expected if only ages 5, 7, 12, and 15 were reported, but it does not considerably alter the distribution of visits in the long term (Supplementary Fig. 3). Looking at the number of visits by age, data shows that most visits reported to SCOR centers around, but falls outside of, the mandatory ages of report, both before and after the implementation in 1993 (Fig. 3). This may suggest that appointments are made based on need, rather than to fulfill criteria for mandatory reporting at a certain age. The Danish Association of Publicly and Privately Employed Dentists (ATO) has noted that there has been a decrease in performed mandatory registrations over the past years, so that in 2018, 25% of mandatory registrations (of children aged 5, 7, 12 and 15) were not done. The ATO cannot ascertain whether this is due to an increase in no-shows at the clinics, or an improvement in oral health status leading to increased intervals between routine examinations [21]. Our data would seem to suggest the latter. If that is the case, one may well question the notion of “mandatory” amongst Danish dentists. Why dentists apparently have not been very strict with following what has been mandatory to report over the years of SCOR is hard to say. To the best of our knowledge there are no consequences of not reporting properly to SCOR, which may well be a factor in the inconsistencies in the reporting of mandatory registrations.

As registration criteria change over datasets, direct comparison of some measures between datasets can be difficult (Tables 1 and 2, Supplementary Tables 1, Table 3). If looking at only one dataset, e.g. 1972–1987, these issues are diminished. For variables not directly comparable across datasets due to changing registration criteria aggregate variables of measurements can be computed in most cases, albeit at the loss of some information. As a proof of principle, we presented examples of how to create aggregate variables for gingivitis, periodontitis, and caries. In accordance with previous studies, females display a marginally higher prevalence of caries [22] and males of gingivitis [23, 24] and periodontitis [25]. Marked spikes can be observed in the prevalence graphs for gingivitis and periodontitis (Fig. 4: Panel A-D). These spikes coincide temporally with changes in registration approaches for both gingivitis and periodontitis (Tables 1 and 2). The prevalence graphs for caries does not display similar spikes, and correspondingly have not undergone significant registration changes since 1972, though the prevalence has changed heavily over time with an almost 50% decrease from 1972 to 2021 (Fig. 4: Panel E-F). It is possible that the criteria for being classified as having gingivitis and periodontitis set up for the purposes of this paper are not well aligned between registration criteria. However, the observed spikes also raises the question of whether gingivitis and periodontitis are subject to general underreporting, or severe overreporting in the years around registration changes, perhaps due to an increased awareness of the diseases caused by the new registration criteria, and a period of calibrating to the new system. Both seem likely. Unfortunately, for periodontitis data collected 1972–1999, and gingivitis data collected after 1988, it is not possible to distinguish between a fully healthy dentition and the registrations simply not having been done at a given visit. In both instances all fields in the registration form pertaining to this disease are left blank (Tables 1 and 2). It is therefore also difficult to estimate the extent to which periodontitis and gingivitis are subject to underreporting based on SCOR data alone.

Comparing the number of registered visits by disease status, both gingivitis and the caries groups are distributed as one may expect based on the idea of visits based on needs. For caries, those with the highest level of disease having the highest mean number of entries into SCOR (Fig. 5: Panel A and C). The average number of visits for individuals with periodontitis fluctuates rather more, sometimes going below the mean number of visits for healthy individuals (Fig. 5: Panel B). This may in part be due to individuals with periodontitis being referred to treatment at specialist clinics outside the DCOHS.

In conclusion SCOR provides a highly detailed source of information on oral health across all birth cohorts from 1965 to the present day. Combined with rich opportunities for linking with data from other Danish registries, cohort studies and more, SCOR provides a unique opportunity to study the impact of childhood oral health on life trajectories of health and disease. However, as described above, using the registry is not without issues. Thus, the available measurements in each dataset should be carefully considered when conceptualizing a research project involving SCOR. This is particularly relevant for the core dental variables caries, gingivitis, and periodontitis, of which we consider caries to be the most reliable measure across SCOR. While we have tried to give a thorough introduction in this paper, we strongly recommend anyone interested in using SCOR to consult with experts in the field of odontology, to ensure an accurate use and interpretation of SCOR data.

### Electronic supplementary material

Below is the link to the electronic supplementary material.

Supplementary Material 1 (Figure 1)

Supplementary Material 2 (Table 1)

Supplementary Material 3 (Figure 2)

Supplementary Material 4 (Figure 3)

Supplementary Material 5 (Table 3)

Supplementary Material 6 (Table 4)

Supplementary Material 7 (Table 2)

Supplementary Materials 8 (Overview)

## Data Availability

The full data from SCOR underlying the present article are not publicly available, but can be used under license from the Danish Health Data Authority [26]. Part of the derived data supporting the conclusions of this article are included within the article and its supplementary files. Other materials such as laws and guidelines for registrations in SCOR drawn upon in this study are publicly available online [11, 12], and from the corresponding author on reasonable request. Note that all material is in Danish. The R script created for cleaning data and generating the analyses presented in the present paper can be found on Github [27].
