# Correction: The national child odontology registry (SCOR): a valuable resource for odontological and public health research

**DOI:** 10.1186/s12903-024-03990-8

**Published:** 2024-02-22

**Authors:** Nikoline Nygaard, Lars Ängquist, Daniel Belstrøm, Evelina Stankevic, Torben Hansen, Anja Olsen, Kasper Rosing, Merete Markvart

**Affiliations:** 1 Department of Odontology, Section for Clinical Oral Microbiology, Faculty of Health and Medical Sciences, University of Copenhagen, Nørre Allé 20, Copenhagen, 2200 Denmark; 2 Novo Nordisk Foundation Center for Basic Metabolic Research, University of Copenhagen, Blegdamsvej 3b, Copenhagen, 2200 Denmark; 3 Danish Cancer Society Research Center, Nutrition and Biomarkers, Strandboulevarden 49, Copenhagen, 2100 Denmark; 4 Department of Odontology, Section for Community Dentistry, Faculty of Health and Medical Sciences, University of Copenhagen, Nørre Allé 20, Copenhagen, 2200 Denmark

**Correction to: Nygaard et al. BMC Oral Health (2023) 23:608**

10.1186/s12903-023-03199-1

In this article [1], there was a mistake pertaining to the index-teeth mentioned in Tables 1 and 2 (first column in both). The index teeth mentioned were “tooth 52 and 55, and 82 and 85 in the temporary dentition, and 12 and 16, and 42 and 46 in the permanent dentition”, where it should have been “tooth 52, 55, and 72 and 75 in the temporary dentition, and 12 and 16, and 32, 36 in the permanent dentition”.
