# Supplementary material for: The national child odontology registry (SCOR): a valuable resource for odontological and public health research
Source: BMC Oral Health. 2023 Aug 29;23:608. doi: 10.1186/s12903-023-03199-1 (PMC10466686; doi:10.1186/s12903-023-03199-1)
Supplement: Supplementary file 1 — Supplementary Material 1 (Figure 1) [file 12903_2023_3199_MOESM1_ESM.pdf]

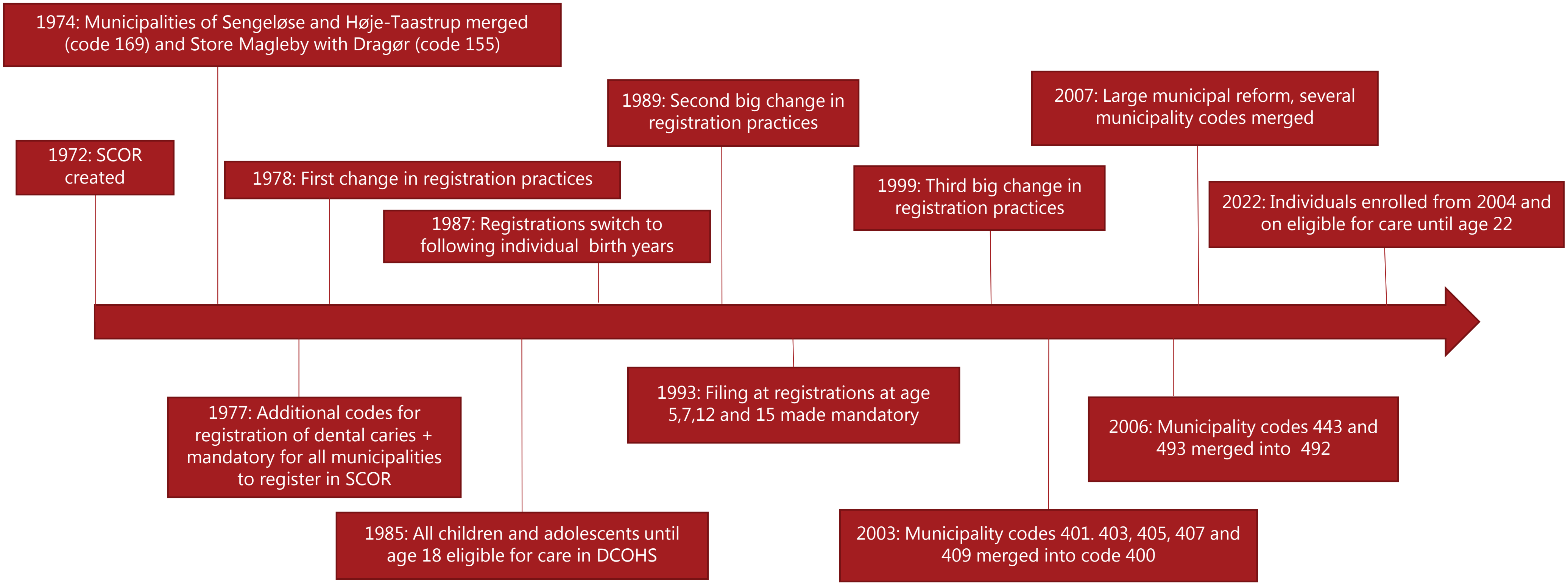

Supplementary figure 1: Timeline of major events relating to SCOR such as changes in registration criteria or changes in the structure of municipalities
