# Supplementary material for: The national child odontology registry (SCOR): a valuable resource for odontological and public health research
Source: BMC Oral Health. 2023 Aug 29;23:608. doi: 10.1186/s12903-023-03199-1 (PMC10466686; doi:10.1186/s12903-023-03199-1)
Supplement: Supplementary file 2 — Supplementary Material 2 (Table 1) [file 12903_2023_3199_MOESM2_ESM.docx]

Supplementary table 1: Registration of dental caries in SCOR, 1972-2022.

|  | 1972-1977 | | 1977-1988 | 1988-1999 | | 2000 - Present | |
| --- | --- | --- | --- | --- | --- | --- | --- |
| Code | Diagnosis | Clinical Findings | Diagnosis | Diagnosis | Clinical Findings | Diagnosis | Clinical Findings |
| Blank | Healthy. | Clean and without discoloration. | =/= | =/= | =/= | =/= | =/= |
| 0 | Initial caries.* | Beginning shadow in the enamel, white dull spots or areas in the enamel without caviation, possibly small lines of discoloration, no changes in enamel hardness on probing. | =/= | Active caries, non-caviated. | The enamel is rough, matte and visibly opaque compared to the surrounding translucent enamel. Symptoms are not combined with caviation. | Initial caries. | The enamel is rough, matte and visibly opaque. Non-caviated. |
| 1 | Primary caries. | Translucent enamel or caviation of the enamel or deeper. With or without discoloration. Changes in enamel or dentin hardness on probing. | =/= | Active caries, caviated. | The enamel is rough, matte and visibly opaque compared to the surrounding translucent enamel. Symptoms are combined with caviation (judged directly by the use of diagnostic tools). | Manifest caries. | The enamel is rough, matte and visibly opaque. With caviation judged directly or using diagnostic tools. Clear shadows beneath seemingly clinically intact enamel (e.g. hidden occlusal caries). |
| 2 | Secondary caries, defect/lost filling. Primary caries on a previously filled surface. Tooth fracture requiring treatment. | N/A | Secondary caries, defect/lost filling, primary caries on a previously filled surface. | Active caries on a previously filled surface, defective/lost filling.** | N/A | Secondary caries. | Manifest caries on a surface previously filled due to caries. |
| 3 | N/A | N/A | Trauma resulting in a fractured tooth, tooth fractures in need of treatment or already treated. | Filling due to trauma. | N/A | Trauma. | Damage caused by acute mechanic trauma of dental hard tissues, pulpa or parodontium. Filling, dental crown or endodontic treatment due to trauma. |
| 4 | Filling of any kind. | N/A | =/= | Filling.** | N/A | Filling. | All fillings and dental crowns, made due to caries. |
| 5 | N/A | N/A | =/= | Endodontically treated tooth, caries causa. | N/A | Endodontically treated tooth. | Pulp capping class 1 or 2, or root canal treatment caries causa, only done on the occlusal (oral) surface. |
| 6 | Extraction caries causa. | N/A | =/= | Extraction caries causa. | N/A | Extraction caries causa. |  |
| 7 | Lost due to other causes (trauma, ortodontics). | N/A | =/= | Lost due to other cause. | N/A | Lost (due to other cause). | E.g. due to trauma or ortodontic treatment. |
| 8 | N/A | N/A | N/A | Fissure-sealed tooth. | Is registered if the groves on the given surface is covered by sealant material. This is a non-mandatory registration. Code 8 can also be used to register other conditions based on the needs/wishes of the clinic. | Chronic caries.* | Previously applied fissure sealant. The sealant material has to cover all or parts of the groves on a given tooth. |
| 9 | N/A | N/A | N/A | Inactive caries.* | The enamel surface is hard, smooth and shiny, but white spots can be observed. In older lesions possibly brown-ish pigmented areas. The symptoms can be accompanied by wearing of the tooth surface and/or caviation. | N/A | N/A |
| Notes | Cases of caries or a filling on the incisal edge are registered on the buccal surface. | |  |  | | Incisals are not considered independent surfaces, and cavities or fillings on these are registered as being on the lingual surface of the given tooth. | |

*Non-mandatory registration
**When a filling involves a non-caviated surface this surface is also registered as filled with code 2 or 4 if it is an approximal or occlusal filling for a fissure on the facial or lingual surface. A non-caviated facial or oral surface that is included the preparation remains.
