# Supplementary figures and images for: The national child odontology registry (SCOR): a valuable resource for odontological and public health research
Source: BMC Oral Health. 2023 Aug 29;23:608. doi: 10.1186/s12903-023-03199-1 (PMC10466686; doi:10.1186/s12903-023-03199-1)

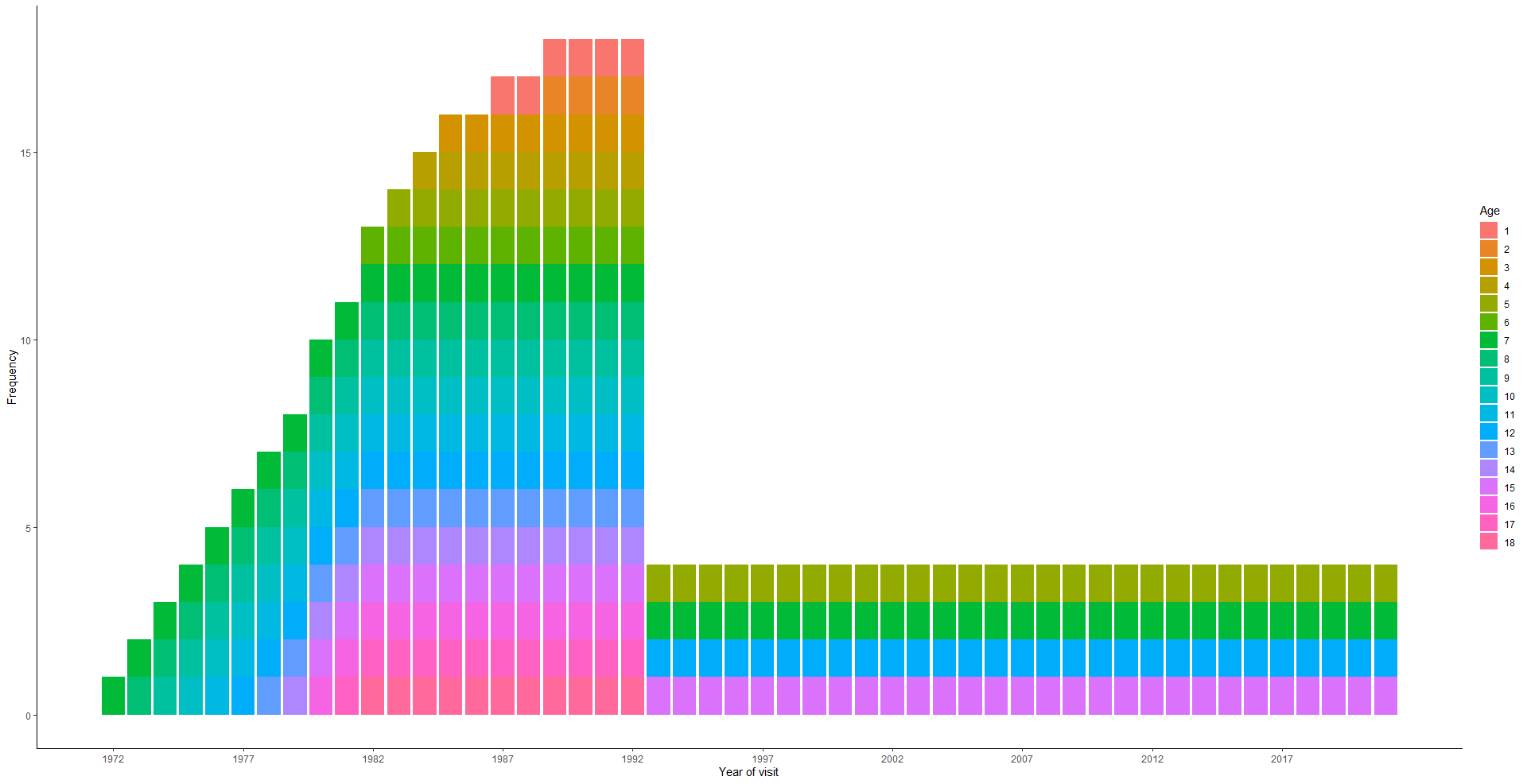

Supplement: Supplementary file 3 — Supplementary Material 3 (Figure 2) [file 12903_2023_3199_MOESM3_ESM.png]

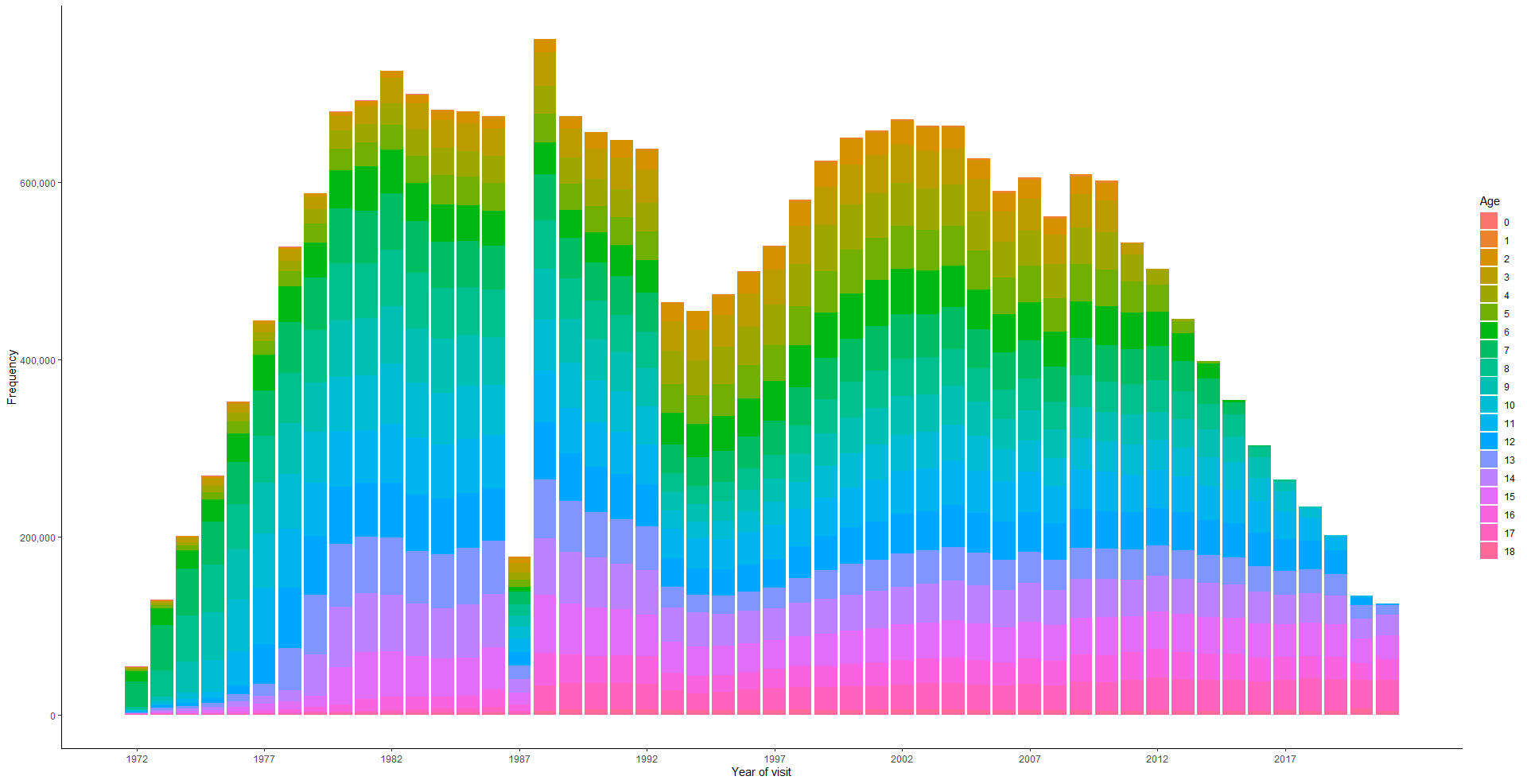

Supplement: Supplementary file 4 — Supplementary Material 4 (Figure 3) [file 12903_2023_3199_MOESM4_ESM.png]
