# Supplementary material for: The national child odontology registry (SCOR): a valuable resource for odontological and public health research
Source: BMC Oral Health. 2023 Aug 29;23:608. doi: 10.1186/s12903-023-03199-1 (PMC10466686; doi:10.1186/s12903-023-03199-1)
Supplement: Supplementary file 8 — Supplementary Materials 8 (Overview) [file 12903_2023_3199_MOESM8_ESM.docx]

**Supplementary Files**

**Supplementary Figure 1**

File format: pdf

Title: SCOR Timeline

Description: A timeline of major events relating to SCOR such as changes in registration criteria or changes in the structure of municipalities.

**Supplementary figure 2**

File format: png

Title: Theorized distributioon of registrations in SCOR

Description: A figure of the theoretical distribution of registrations in SCOR based on guidelines as described in the text. Please note that the y-axis values do not reflect the actual number of enrolled individuals, but equals height one for all single combinations of age and year.

**Supplementary figure 3**

File format: png

Title: Empirical distribution of registrations in SCOR

Description: A figure of the empirical distribution of registrations in SCOR. See Supplementary table 2 for the numbers underlying the figure.

**Supplementary Table 1**

File format: docx

Title: Caries registration in SCOR

Description: An overview of how dental caries have been registered in SCOR in the period 1972-2022.

**Supplementary table 2**

File format: xlsx

Title: Visits by age and year of visit

Description: Full data on the number of visits at a given age by year of visit.

**Supplementary table 3**

File format: xlsx

Title: Comparing SCOR and DST data

Description: Full data on the difference in number of individuals registered in SCOR by birth year, and the yearly number of live births and immigrants granted citizenship according to DST data.

**Supplementary table 4**

File format: xlsx

Title: Visits by age and year of report

Description: Full data on the number of visits at a given age by year of report.
